# A Rare Case of an Infectious Pseudoaneurysm due to Aspergillus flavus in the Setting of Renal Transplant

**DOI:** 10.7759/cureus.4208

**Published:** 2019-03-09

**Authors:** Samia Asif, Joseph Bennett, Rebecca R Pauly

**Affiliations:** 1 Internal Medicine, University of Missouri, Kansas City, USA; 2 Internal Medicine, Virginia Tech-Carilion School of Medicine, Roanoke, USA

**Keywords:** aspergillus flavus, renal transplant, pseudoaneurysm

## Abstract

Renal transplant as a treatment option for end-stage renal disease (ESRD) is becoming increasingly prevalent. As with any other surgical intervention, complications may occur, including vascular ones. Pseudoaneurysms are particularly rare, with mycotic aneurysms reported in less than 1% of patients after renal transplant. Here, we present a case of an infected pseudoaneurysm involving the renal artery anastomosis, resulting in the explantation of the transplanted kidney.

A 77-year-old man underwent deceased donor renal transplant for ESRD in the setting of diabetes and hypertension. He presented with acute kidney injury; a renal biopsy revealed mild active cellular rejection, treated with high-dose steroids. As renal function continued to deteriorate, a repeat renal biopsy was performed. During ultrasound-guided biopsy of the transplanted kidney, a possible aneurysm proximal to the anastomosis of the renal artery to the right external iliac artery was seen. A pelvic arteriogram showed a large 3 cm x 3.4 cm x 4 cm pseudoaneurysm arising directly off the right external iliac artery with the renal transplant artery filling from the distal side of this aneurysm. The patient was taken to the operation room for a re-exploration of his transplanted kidney and revision of the arterial anastomosis. Intraoperatively, necrotic tissue and purulence within the pseudo-aneurysm were noted with failure to salvage blood supply to the transplanted kidney; both the infected pseudo-aneurysm and renal transplant were resected. A portion of the aneurysm was sent to microbiology for culture; fungal cultures grew Aspergillus flavus. He was treated with isavuconazonium and improved clinically, though he subsequently expired following a sudden cardiac arrest.

Given its rarity, most medical professionals will be unfamiliar with this unusual complication in renal transplant patients. Our case highlights the importance of pursuing imaging modalities to help identify vascular complications and discusses how to proceed after diagnosis is made. The importance of knowing this process is paramount in improving future patient outcomes.

## Introduction

Renal transplant remains the preferred treatment modality for end-stage renal disease (ESRD), particularly given the improvements in surgical techniques and immunosuppressive therapies. Common complications include acute or chronic graft rejection and nephropathy secondary to immunosuppressive agents. More frequently seen vascular complications include renal arterial stenosis, followed by renal arterial or venous thrombosis [1]. Pseudoaneurysms are particularly rare, with mycotic aneurysms reported in less than 1% of patients who undergo renal transplant [2]. Given its rarity, most medical professionals will be unfamiliar with this complication. This results in a low suspicion for the diagnosis and, hence, no pursuit of imaging modalities that will help identify this complication, leading to adverse patient outcomes. Moreover, many providers may be inexperienced with the critical actions of management once the diagnosis is made. It is important to highlight this case to help guide in the early recognition of this rare complication that can lead to deleterious patient outcomes. Here, we present a case of an infectious pseudoaneurysm in a renal transplant patient, which resulted in transplant nephrectomy.

## Case presentation

A 77-year-old man with a past medical history of type 2 diabetes, hypertension, and ESRD underwent deceased donor renal transplantation. Two months following his renal transplant, the patient was admitted for an acute kidney injury discovered on routine follow-up laboratory testing. From a baseline creatinine of 1.3 mg/dl after the transplant, he was noted to have a creatinine of 2.7 mg/dl and a renal biopsy was obtained. Histopathology was suggestive of mildly active cellular rejection and acute tubular injury but with no concerns for antibody-mediated rejection. The patient received three days of methyl-prednisolone 250 mg daily intravenously and was subsequently transitioned to high-dose oral prednisone with a taper.

**Figure 1:**
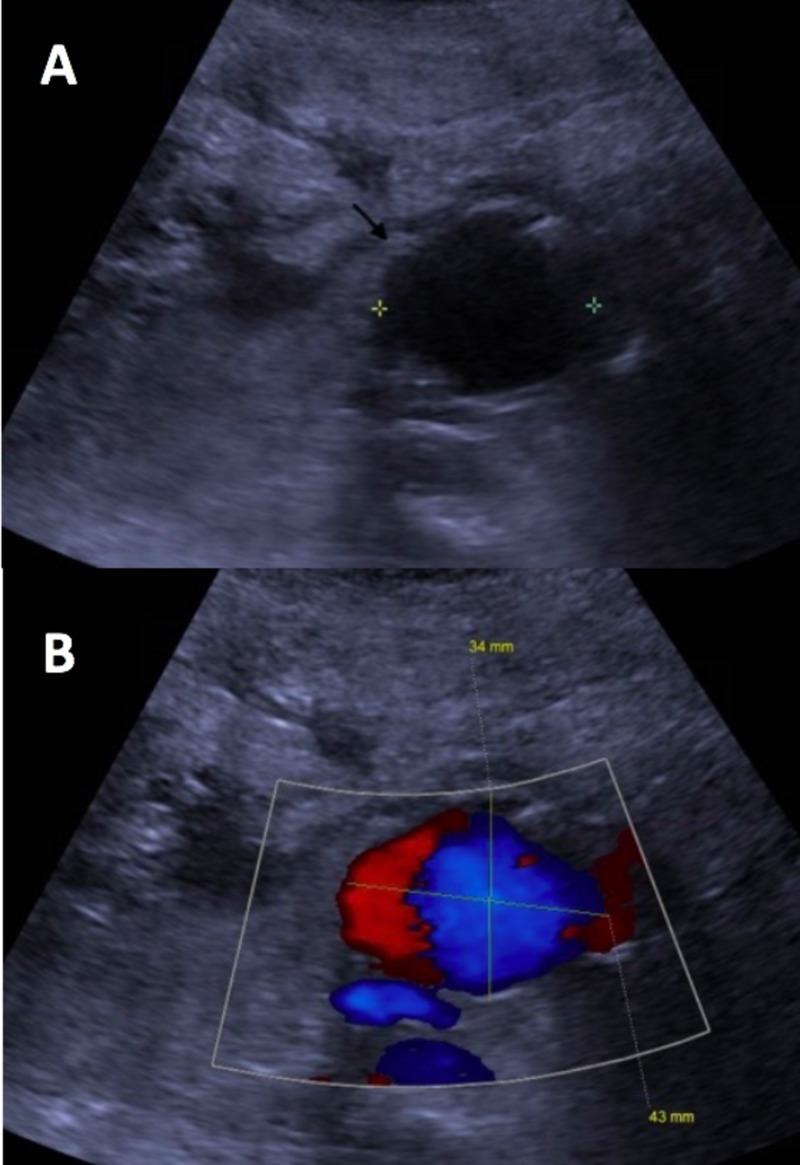
Ultrasound image of transplant kidney showing: (a) a rounded hypo-echoic area adjacent to the transplant kidney; (b) confirmed by color Doppler to be a pseudoaneurysm

The patient was readmitted within three weeks of his renal biopsy with worsening renal functions, now with a creatinine level of 3.8 mg/dl. At this time, he was on prednisone five mg daily, tacrolimus four mg twice daily, and mycophenolate 500 mg twice daily. His family reported poor oral intake and that he had been taking furosemide at home. He received intravenous (IV) normal saline and diuretics were held. However, as his renal functions did not improve beyond a creatinine level of 2.1 mg/dl, the decision was made to perform a repeat renal biopsy. During ultrasonography for the renal biopsy, concerns were raised for a possible renal artery aneurysm. An ultrasound of the right lower quadrant and transplant kidney subsequently showed a 3 cm x 3.4 cm x 4 cm aneurysm proximal to the renal artery anastomosis to the right external iliac artery (Figure 1). The right external iliac artery to renal artery anastomosis was patent and the renal vein was noted to be patent as well. No peri-transplant kidney fluid collections were noted and no hydronephrosis was seen.

**Figure 2:**
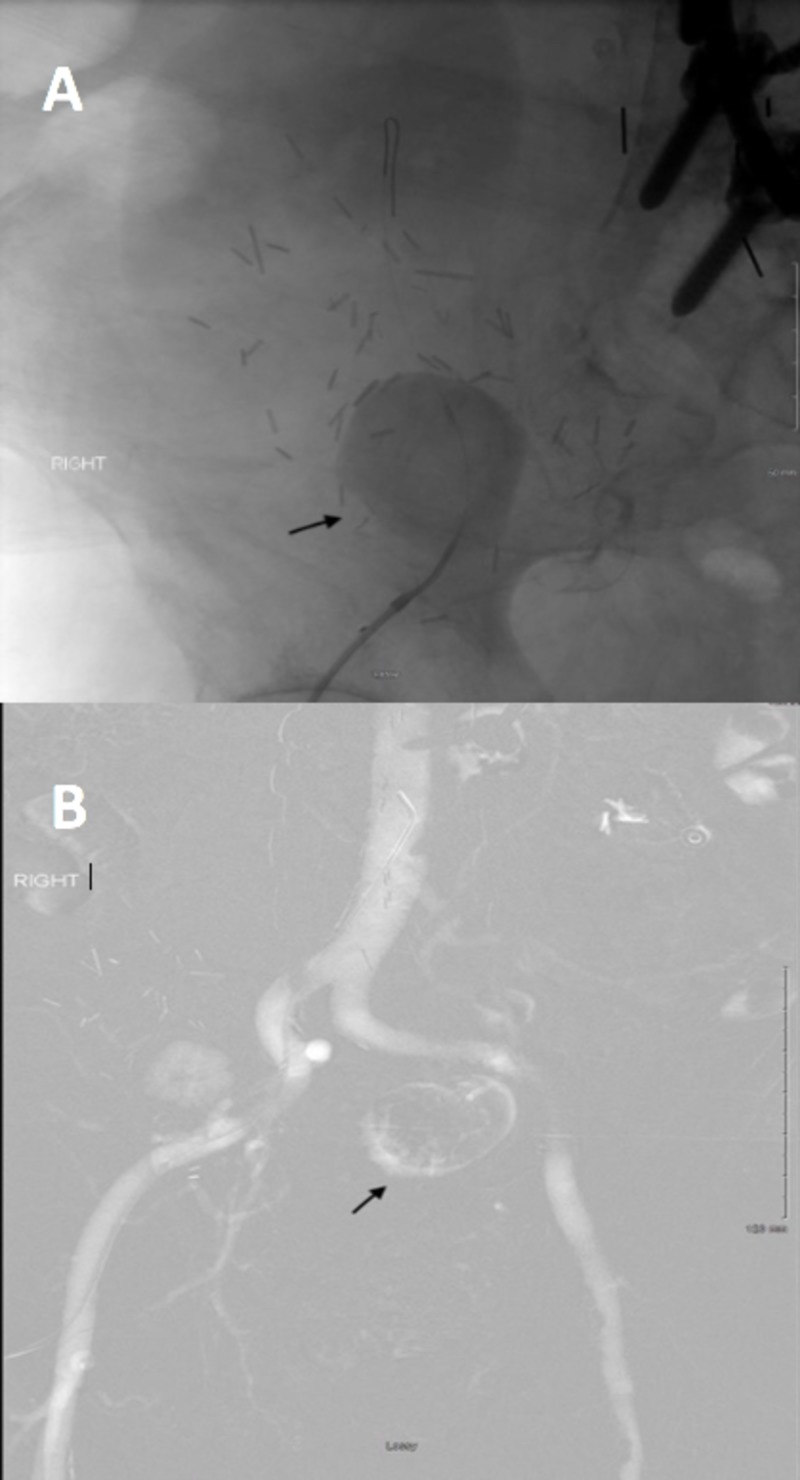
Arteriogram of transplant kidney showing a pseudoaneurysm arising at the anastomotic site

Subsequently, a pelvic arteriogram was performed, which showed patent pelvic and iliac arterial flow. However, a large pseudoaneurysm arising directly off the right external iliac artery was re-noted and the renal transplant artery was noted to be filling from the distal side of the aneurysm and remained patent, although with sluggish flow (Figure 2). A second known renal transplant artery was not seen and was presumed to be thrombosed.

While the interventional radiologist considered closing off the pseudoaneurysm by stent placement, the origin of the aneurysm was noted to be too close to the anastomosis to allow for stent placement. Hence, transplant surgery was consulted. The patient was taken to the operation room for a re-exploration of his transplanted kidney and revision of the arterial anastomosis. The patient underwent exploratory laparotomy, and intraoperatively, significant inflammation and scar tissue surrounding the hilum of the transplanted kidney was seen. Once the pseudoaneurysm was entered, necrotic tissue and purulence within the pseudoaneurysm were noted. It was felt that the arterial flow to the transplanted kidney could not be restored and, therefore, a decision was made to proceed with the resection of the infected pseudoaneurysm and transplant nephrectomy. The transplanted kidney was explanted. A portion of the pseudoaneurysm was sent to pathology for further characterization, and a portion was sent to microbiology for culture. The explanted kidney was also sent to pathology for further analysis. The patient was transferred to the intensive care unit (ICU) postoperatively for further management and close monitoring. During the surgery, the patient received crystalloids and multiple blood products.

On arrival to the ICU, the patient had temporary dialysis access placed and started on continuous renal replacement therapy (CRRT). Given concerns for an infected pseudoaneurysm, he was empirically started on renally dosed vancomycin, piperacillin-tazobactam, and fluconazole. All immunosuppressive agents were discontinued at this time. Due to persistent vasopressor requirements while in the ICU, the patient was switched to vancomycin, meropenem, and micafungin. The infectious disease team was consulted for recommendations. Blood bacterial and fungal cultures remained negative. However, fungal cultures from the pseudoaneurysm grew Aspergillus flavus on three separate samples. The patient was then transitioned to IV isavuconazonium 372 mg every eight hours for six doses, followed by 372 mg daily with a plan to continue for at least six weeks. Vancomycin and meropenem were discontinued. The pathology of the explanted kidney showed transmural necrosis of the renal artery, no evidence of rejection, and scattered micro-abscesses within the parenchyma. As the patient improved clinically, he was transitioned from CRRT to intermittent hemodialysis. He was subsequently transferred out of the ICU in stable condition. His hospitalization was complicated by colitis secondary to Clostridium difficile for which oral vancomycin was initiated. Preparation was made to discharge him to an acute rehabilitation facility; however, a day prior to discharge, he was found to be unresponsive by his nurse and despite prolonged resuscitation attempts for cardiac arrest, could not be revived. The exact etiology for this sudden demise could not be determined.

## Discussion

Infectious pseudoaneurysms are a rare entity with potentially devastating consequences. Transplant recipients are susceptible to such infectious complications because they are maintained on immunosuppressive agents. Chung et al. summarized 30 cases of infectious pseudoaneurysms after a review of the literature, clinical presentation, culprit organism, and modality of treatment. Candida was reported in 12 cases, with identification as Candida albicans (C. Albicans) in 11 of these. Aspergillus species was cultured in 10 of the 30 cases. Pseudomonas aeruginosa was found in four cases. Methicillin-resistant Staphylococcus aureus (MRSA) was detected in one case. All except four cases resulted in graft nephrectomy. The MRSA case was the only one in which antibiotic therapy was used independently of any surgical intervention. Other treatment modalities included: excision of pseudoaneurysm with prosthetic graft reconstruction (C. albicans), a balloon-expandable covered stent (Klebsiella), and a balloon-expandable covered stent with aneurysmectomy with femoral-femoral bypass (Mucorales) [3].

Mycotic aneurysms should be suspected in a patient with renal transplant who presents with hemorrhagic shock, fever with unclear etiology, anemia, abdominal pain, or a pulsatile mass. Rarely, mycotic aneurysms may present with acute kidney injury or patients may be asymptomatic [4]. Due to a myriad of clinical presentations, a high index of suspicion is required to make the diagnosis. The rupture of these aneurysms is a dangerous but well-recognized complication [5]. Fungal agents comprise 5% of infections in renal transplant cases and commonly occur within the first six months of transplant, likely correlating with concomitant immunosuppression [6].

For diagnostic workup, color-coded Doppler ultrasonography is a good initial test to identify a pseudoaneurysm. This can subsequently be confirmed with fluoroscopy, computed tomography (CT), or magnetic resonance angiography (MRA). Treatment for each case has to be individualized, but early diagnosis and management may allow the preservation of a renal allograft.

## Conclusions

All medical practitioners frequently encounter acute renal injuries. It is essential to have an increasing awareness regarding the possible etiologies of acute kidney insults. Increased cognizance of such rare but emergent complications witnessed in renal transplant recipients will allow for appropriate early management or, at least, a timely referral made to a relevant specialty such as infectious disease or surgery. In doing so, a misdiagnosis such as renal dysfunction attributed initially to poor volume status can be avoided and better patient outcomes achieved.

## References

[1] Rijksen JF, Koolen MI, Walaszewski JE, Terpstra JL, Vink M (1982). Vascular complications in 400 consecutive renal allotransplants. J Thorac Cardiovasc Surg.

[2] Ministro A, Ferreira T, Batista L, Santana A, Alves N, Guerra J, Fernandes e Fernandes J (2017). Mycotic pseudoaneurysm after kidney transplantation: two case reports. Transplant Proc.

[3] Chung MMT, Chan YC, Law Y, Cheng SW (2017). Infectious anastomotic pseudoaneurysm complicating renal allograft: case report and review of literature. Int J Nephrol Renovasc Dis.

[4] McIntosh BC, Bakhos CT, Sweeney TF, DeNatale RW, Ferneini AM (2005). Endovascular repair of transplant nephrectomy external iliac artery pseudoaneurysm. Conn Med.

[5] Jordan ML, Cook GT, Cardella CJ (1982). Ten years of experience with vascular complications in renal transplantation. J Urol.

[6] Fishman JA (2002). Overview: fungal infections in the transplant patient. Transpl Infect Dis.

